# Effect of natural zeolite in diets with or without protein reduction on the performance, carcass and organ yield, and litter quality of broilers

**DOI:** 10.1016/j.psj.2026.106434

**Published:** 2026-01-12

**Authors:** Camila Guedes Valadares, Maria do Carmo Mohaupt Marques Ludke, Jorge Vitor Ludke, Carlos Bôa-Viagem Rabello, Gláucia Manoella de Souza Lima Gomes, Dayane Albuquerque da Silva, Apolônio Gomes Ribeiro, Arlei Coldebella, Esterfani Pereira da Silva, Marcela de Araújo Sobral, Kananda Rohhden dos Santos, Clener Manoel Albino Fausto, Lucas Rannier Ribeiro Antonino Carvalho

**Affiliations:** a Universidade Federal Rural de Pernambuco, Animal Science Department, Recife, Pernambuco, Brazil; b Embrapa Suínos e Aves, Concórdia, Santa Catarina, Brazil; c Universidade Federal de Pernambuco, Department of Biomedicine, Recife, Brazil; d Universidade Federal da Paraíba, Animal Science Department, Areia-PB, Brazil; e Department of Physiology and Pharmacology Karolinska Institutet - Stockholm Sweden Biomedicum, 5B, Solnavägen 9, S-171 77, Stockholm, Sweden

**Keywords:** Broilers, Clinoptilolite, Protein level, Performance, Ammonia

## Abstract

Ammonia emissions from poultry litter represent a major environmental and production challenge in modern broiler systems and are strongly influenced by dietary crude protein levels and nitrogen utilization efficiency. Natural zeolites, particularly clinoptilolite, have been proposed as nutritional strategies to mitigate ammonia volatilization; however, their effects on broiler performance remain inconsistent, especially when combined with protein reduction. This study evaluated the effects of natural zeolite (Celpec®) inclusion at 0, 1.0, 2.0, and 3.0% in diets formulated with two crude protein levels (recommended or reduced by 3%) on growth performance, carcass and organ yield, and litter quality of broiler chickens. A total of 720 one-day-old male Ross broilers were allocated in a completely randomized design in a 4 × 2 factorial arrangement, with six replicates of 15 birds each. Growth performance (feed intake, body weight gain, and feed conversion ratio), carcass yield and commercial cuts, relative weight of digestive organs, and litter quality (in natura ammonia concentration and microbiological evaluation) were assessed. Data were analyzed using analysis of variance, regression analysis, and Dunnett’s test (*P* < 0.05). No significant interaction between zeolite inclusion level and dietary crude protein was observed. Zeolite inclusion above 1% impaired growth performance, particularly in diets with reduced crude protein. Crude protein reduction alone negatively affected performance from 35 d of age onward. Zeolite inclusion did not reduce Escherichia coli counts in the litter. However, inclusion of up to 1% zeolite reduced ammonia concentration in the litter without compromising growth performance or carcass yield, regardless of dietary protein level. These findings indicate that dietary inclusion of clinoptilolite at 1% is an effective strategy to reduce ammonia emissions from broiler litter without impairing performance or carcass yield, provided that diets are formulated with adequate protein and amino acid balance, whereas higher inclusion levels or protein restriction compromise productive responses.

## Introduction

In recent years, consumers have become increasingly concerned about food production processes, especially regarding the adoption of sustainable practices that minimize impacts on the environment and on human and animal health (Upright, 2023; Ammann et al., 2024). The poultry production chain, although efficient, is responsible for the generation of waste that, if managed inadequately, can compromise the sustainability of the system. Nitrogen excretion by birds is considered one of the main environmental challenges, since a significant part of the nitrogen ingested is not converted into animal products, and is excreted mainly in the form of uric acid, which decomposes into ammonia (Sousa et al., 2016).

Ammonia (NH₃) emissions in poultry facilities are directly associated with the quality of the diet, especially crude protein (CP) levels. Nutritional strategies, such as reducing CP with industrial amino acid supplementation, have shown potential to reduce these emissions, in addition to promoting benefits to bird performance (Tang et al., 2023). At the same time, nutritional additives can contribute to the modulation of the intestinal microbiota and reduction of ammonia production, promoting a healthier breeding environment (Prasai*.,* 2017).

Environments with high ammonia levels (>25 ppm) compromise the integrity of the respiratory epithelium and favor infections, while exposures above 50 ppm reduce weight gain and worsen feed conversion (Orffa, 2020). At 100 ppm, there is worsening of lung lesions and an increase in the release of inflammatory cytokines (Chen et al., 2022). Therefore, countries with advanced poultry farming recommend strict limits: the European Union and Australia indicate values <20 ppm, and OSHA, in the USA, establishes 25 ppm as the occupational limit (Csiro, 2019; Ohio State University, 2020). Among the mitigation strategies, the use of zootechnical additives with adsorbent capacity stands out. They act in the retention of nitrogen compounds in the excreta (Oliveira, 2021).

In this context, natural zeolites, especially clinoptilolite, have been studied for their adsorption and ion exchange capacity. These minerals can contribute to improving the poultry environment by reducing excreta moisture, retaining ammonia, and promoting intestinal health, with beneficial effects on performance (Reis et al., 2020; Ezenwosu et al., 2022; Elsherbeni et al., 2024). However, the results in literature are still contradictory regarding the effects of zeolite on growth performance, and on the reduction of ammonia emissions, especially due to variations in the composition and purity of the product (Eleroglu et al., 2011; Tatar et al., 2012; Schneider et al., 2016).

Associating clinoptilolite to diets with marginal protein reduction for broilers still lacks studies, but it is a promising strategy to combine environmental sustainability and productive performance. Therefore, the present study aimed to evaluate the effect of including clinoptilolite zeolite (0, 1, 2, and 3%), with and without marginal reduction of crude protein, on the performance, carcass and commercial cut yield, relative weight of organs, and quality of broiler litter.

## Material and methods

The experimental protocol was approved by the Animal Ethics Committee (CEUA) of the Federal Rural University of Pernambuco, Brazil, under approval certificate Number 9008090921.

### Installations, animals, and experimental diets

The study was carried out in the city of Recife, state of Pernambuco, Brazil (8°01′17.4" S, 34°57′14.8" W, and altitude of 15 m). A total of 720 1-day-old male broiler chicks of the Ross lineage with an average initial weight of 47±3 g, were used. The birds were housed in a masonry shed divided into boxes measuring 1.15 m x 1.80 m each, with screened sides and a masonry floor covered with wood shavings, and equipped with a nipple drinker and a tubular feeder.

The birds were distributed in a completely randomized design, in a 4 × 2 factorial scheme, corresponding to four levels of inclusion of clinoptilolite zeolite (Celpec®) in their diet (0, 1, 2, and 3%), and two levels of crude protein (adequate/recommended – REC and reduced by 3% – RED), totaling eight treatments. The choice of the 3% protein reduction was based on previous studies that demonstrate the feasibility of minimizing the protein content of the diets without compromising growth performance, when associated with the use of additives that favor the digestibility of nutrients. Each treatment consisted of six replicates, with 15 birds per experimental unit.

Eight experimental diets were formulated in four phases (1 to 7; 8 to 21; 22 to 35, and 36 to 42 days of age), based on corn, soybean meal, and meat and bone meal, with phosphorus and calcium supplementation. The control diet was formulated to meet the nutritional requirements of the birds, as recommended by Rostagno et al. (2017), while the diets with reduced crude protein were formulated with a 3% decrease in CP content in relation to the requirement for each phase (Table 1). In the treatments with protein reduction, the first three limiting amino acids (lysine, methionine+cystine, and threonine) were also adjusted proportionally, respecting an overall protein reduction, without correction with synthetic amino acids. The feed and water were provided *ad libitum* in each experimental unit. The zeolite used belongs to the clinoptilolite group, composed mainly of SiO₂ and Al₂O₃. The chemical and physical composition of the zeolite is described in Table 2.

**Table 1:** Ingredient composition and calculated nutritional values of the basal experimental diets (Z0) for broilers from 1 to 42 days of age.

	Growth phase							
	1-7 days		8-21 days		22-35 days		36-42 days	
Treatments								
	Z0REC	Z0RED	Z0REC	Z0RED	Z0REC	Z0RED	Z0REC	Z0RED
**Ingredients (kg)**								
Corn (6.52%)	48.77	50.93	48.39	50.49	56.66	58.66	61.69	63.49
Soybean meal (45%)	36.14	34.34	35.70	33.92	25.53	23.84	24.14	22.63
Meat and bone meal (41%)	5.99	6.03	5.25	5.29	7.33	7.38	3.39	3.42
Soybean oil	4.26	3.90	5.86	5.51	5.43	5.09	5.94	5.64
Inert^1^	3.00	3.00	3.00	3.00	3.00	3.00	3.00	3.00
Common salt	0.45	0.45	0.45	0.46	0.37	0.37	0.43	0.43
Dl-Metionine 99%	0.44	0.43	0.43	0.41	0.41	0.39	0.33	0.31
Vitamin-mineral premix^2^	0.40	0.40	0.40	0.40	0.40	0.40	0.40	0.40
L-Lysine HCL 78.8%	0.35	0.35	0.31	0.31	0.40	0.41	0.36	0.36
L-Threonine 98.5%	0.17	0.16	0.17	0.16	0.19	0.18	0.14	0.13
BHT^3^	0.01	0.01	0.01	0.01	0.01	0.01	0.01	0.01
Limestone	0.01	0.00	0.02	0.02	0.25	0.26	0.16	0.15
Zeolite	0.00	0.00	0.00	0.00	0.00	0.00	0.00	0.00
Total	100	100	100	100	100	100	100	100
**Nutritional Composition**								
Metabolizable energy, kcal/kg	3000	3000	3100	3100	3200	3200	3250	3250
Crude protein %	22.50	21.83	21.93	21.27	20.45	19.84	17.67	17.14
Ether extract %	7.34	7.03	8.84	8.54	8.76	8.71	8.62	8.58
Crude fiber %	2.78	2.74	2.75	2.70	2.61	2.59	2.53	2.50
Calcium %	1.09	1.09	0.97	0.97	0.86	0.86	0.69	0.69
Available phosphorus %	0.48	0.48	0.43	0.43	0.38	0.38	0.31	0.31
Sodium %	0.23	0.23	0.23	0.23	0.21	0.21	0.20	0.20
**Digestible Amino Acids (%)**								
Digestible lysine%	1.36	1.32	1.30	1.27	1.23	1.20	1.07	1.03
Digestible methionine + cysteine%	0.99	0.96	0.97	0.93	0.91	0.88	0.79	0.77
Digestible threonine%	0.88	0.85	0.86	0.83	0.81	0.79	0.70	0.68

ZxREC = Diets with adequate crude protein levels (REC), with inclusion of 0 (Z0), 1 (Z1), 2 (Z2), or 3% (Z3) zeolite.

1 Inert ingredient: washed sand, included inversely proportional to zeolite to total 3% in the formulation (zeolite + inert);

2 Guarantee levels per kg of product: Iodine (min) 265 mg/kg; Selenium (min) 80 mg/kg; Copper (min) 3,000 mg/kg; Iron (min) 9,550 mg/kg; Manganese (min) 13.5 g/kg; Zinc (min) 12.5 g/kg; Folic acid (min) 250 mg/kg; Niacin (min) 7,800 mg/kg; Biotin (min) 13.9 mg/kg; Pantothenic acid (min) 5,180 mg/kg; Vitamin A (min) 2,400,000 IU/g; Vitamin B1 (min) 550 mg/kg; Vitamin B12 (min) 3.75 mg/kg; Vitamin B2 (min) 1,400 mg/kg; Vitamin B5 (min) 615 mg/kg; Vitamin D3 (min) 590,000 IU/g; Vitamin E (min) 4,250 IU/g; Vitamin K (min) 875 mg/kg; Choline (min) 69.5 g/kg; BHT (min) 100 mg/kg; Halquinol (min) 7,500 mg/kg;

3 Antioxidant.

**Table 2:** Chemical and physical composition of clinoptilolite zeolite Celpec©.

**Chemical Composition (%)**		
**Mineral Oxide**	**Maximum Value**	**Minimum Value**
SiO_2_	75	62
Al_2_O_3_	15	7
Fe_2_O_3_	3	0.50
TiO_2_	0.50	0
CaO	5	0.50
MgO	3	0
Na_2_O	5	0
K_2_O	5	0.50
MnO	0.50	0
P_2_O_5_	0.50	0
ZnO	0.50	0
CuO	0.10	0
**Physical Composition**		
**Analysis**	**Specification**	
Particle size (mesh)	#325 (Opening 0.044 mm)	
Color	Beige to slightly greenish	
Fusion point	1000 a 1400°C	
Bulk density	0.5 – 1.0 g/cm³	
pH	6.5 – 10.0	
Moisture	≤ 6 %	
Cation exchange capacity (CEC)	1.2 a 2.0 mEq/g	

*Manufacturer: Celta Brasil.

### Growth performance

The performance parameters evaluated were: feed intake, weight gain, and feed conversion. The evaluations were performed at the end of each phase (1 to 7 days, 8 to 21 days, 22 to 35 days, and 36 to 42 days of age). Mortality was recorded daily throughout the experimental period, and no mortality was observed in any treatment, resulting in a 100% survival rate across all experimental groups.

### Carcass and commercial cut yield, and relative weight of digestive system organs

At 42 days of age, two birds from each replicate were selected based on the average weight of 3776±199. After fasting for 12 hours, the birds were weighed and euthanized by stunning followed by bleeding.

Carcass yield was calculated based on the weight of the eviscerated carcass (without feet, head, and neck) relative to the fasted live body weight of the birds. Commercial cut yields were calculated using the weights of boneless and skinless breast, legs (thigh + drumstick), and wings, expressed as a percentage of fasted live body weight. Abdominal fat was defined as the fat deposited around the cloacal bursa, gizzard, proventriculus, and adjacent abdominal muscles, and its yield was also expressed relative to fasted live body weight.

Subsequently, the digestive system organs (proventriculus, gizzard, liver, pancreas, and intestine) were collected and weighed on a precision scale (0.0001 g) to determine the relative weight of each organ in relation to fasted live body weight. The intestine was divided into segments, which were measured with a tape measure to obtain the length of the various portions of the intestinal tract (duodenum, jejunum, ileum, cecum, and colon-rectum).

### Litter quality

Approximately 500 g of litter samples were collected per experimental unit (box) at 35 and 42 days for ammonia determination. The method consisted of collecting samples at five different points in the box, avoiding areas close to drinkers and feeders. Ammonia nitrogen analysis of the litter was performed by weighing 100 g of the sample *in natura*, placing it in a 750 mL glass bottle with a lid. Subsequently, a 50 mL beaker containing 10 mL of a 2% boric acid solution was placed over the litter sample inserted in the container to fix the ammonia released by the litter. Immediately after, the containers were closed and left for 17 hours. The determination of the amount (mg) of ammonia (NH_3_) fixed in the boric acid solution was performed by titration with standardized sulfuric acid at a concentration of 0.05, in which the flasks were opened and the beaker with the boric acid solution was removed to perform the titration. The results were expressed in milligrams of ammonia released, by using the formula $A=\frac{vt\;x\;N\;x\;1,7\;}{P}$ where A represents the ammonia concentration (mg of NH_3_); Vt is the volume of the H_2_SO_4_ solution used in the titration (mL); N is the normality of the acid used; and P is the weight of the sample (g) (Hernandes and Cazetta, 2001).

For the quantification of *Escherichia coli*, the material was collected, approximately 50g of sample from the litter per rep/treatment, on 42 days in 5 different points of the litter. Subsequently, the material was homogenized and a fraction equivalent to 1g of the material was removed, which was added to sterilized tubes with 9 ml of peptone water. The material was packaged and transported to the microorganism genetics laboratory (LABGEN) at UFPE. Subsequently, 0.1ml aliquots were removed from the samples and diluted (10-1 to 10-11). 100uL of the dilutions were inoculated with the aid of a Drigalski loop in Petri dishes with 10 ml in BEM agar medium (Eosin Methylene Blue Culture Medium) for E. coli (BAM/FDA: 2016).

The contents of the litter were thawed and serially diluted in saline solution to a concentration of 10-9 to enable identification and counting of the bacteria (Colony Forming Unit, CFU). The methodology of the protocols mentioned above was then followed. The seeded plates underwent an incubation period of 24 hours at 37°C, followed by reading to verify the growth of characteristic colonies according to the bacteria investigated and CFU count.

### Statistical analysis

The data were subjected to analysis of variance (ANOVA) using the F test with ɑ = 0.05 and using the SAS software (Statistical Analysis System, version 9.4) (SAS Institute Inc. 2013). After ANOVA, regression analyses were performed according to the levels of zeolite added, if the F test was significant, using the PROC GENMOD procedure of the same software, to evaluate linear, quadratic, and cubic effects. In addition, the Dunnett's mean test was performed at 5% significance.

The statistical model applied was the following:

$$Y_{\text{ijk}}=\mu +Z_{i}+P_{j}+{\left(\text{ZP}\right)}_{\text{ij}}+e_{\text{ijk}}$$

in which Y_ijk_ is the variable observed in the k-th repetition of the treatment with the i-th level of zeolite and the j-th level of protein; vμ is the general average; Z_i_ is the fixed effect of the zeolite level (i = 1, 2, 3, 4); P_j_ is the fixed effect of protein level (j = 1, 2); (ZP)_ij_ is the effect of the interaction between the zeolite and protein levels; and eijk is the normally distributed random experimental error with mean zero and variance σ2 [eijk ∼ N (0, σ2)] associated with the observation Y_ijk_.

## Results

### Growth performance

There was no interaction between the protein levels of the diets and zeolite levels for any of the variables studied at any of the ages evaluated. However, there was an effect when evaluating the factors separately. Regarding the zeolite levels, there was a significant effect (*P* ≤ 0.05) on weight gain (WG) and feed conversion (FCR) in the phases from 1 to 35 days (*P* = 0.049 and *P* = 0.050) and from 1 to 42 days (*P* = 0.0397 and *P* = 0.0482), respectively (Table 3). When performing regression analysis on the above parameters and phases, a decreasing linear effect (*P* ≤ 0.05) was observed when zeolite was added (Table 8).

**Table 3:** Average values of body weight gain, feed intake, and feed conversion ratio of broilers fed diets with zeolite inclusion with or without protein reduction.

**Phase 1–7 days**											
Parameter	Zeolite (%)							*p-value*			
	0	1	2	3	Mean	Mean CP REC	Mean CP RED 3%				
								Zeólite	CP	Zeo x CP	Regression
BWG	141 ± 2.3	141 ± 1.4	141 ± 1.4	138 ± 2.0	140	141 ± 1.2	140 ± 1.4	0.24	0.29	0.99	NS
FI	157 ± 2.3	155 ± 1.7	156 ± 1.8	154 ± 3.7	155	154 ± 1.6	157 ± 2.8	0.95	0.15	0.27	NS
FCR	1.11 ± 0.03	1.10 ± 0.02	1.10 ± 0.04	1.12 ± 0.03	1.11	1.09 ± 0.01	1.12 ± 0.02	0.72	0.08	0.31	NS
**Phase 1–21 days**											
Parameter	Zeolite (%)							*p-value*			
	0	1	2	3	Mean	Mean CP REC	Mean CP RED 3%				
								Zeólite	CP	Zeo x CP	Regression
BWG	1063 ± 19.4	1057 ± 12.4	1054 ± 14.9	1026 ± 18.3	1050	1053 ± 10.6	1048 ± 13.2	0.06	0.48	0.52	NS
FI	1267 ± 11	1260 ± 13	1258 ± 31	1228 ± 12	1253	1254 ± 9	1252 ± 17	0.18	0.85	0.61	NS
FCR	1.19 ± 0.03	1.19 ±0.03	1.19 ± 0.05	1.19 ±0.04	1.19	1.19 ±0.02	1.19 ± 0.03	0.16	0.47	0.33	NS
**Phase 1–35 days**											
Parameter	Zeolite (%)							*p-value*			
	0	1	2	3	Mean	Mean CP REC	Mean CP RED 3%				
								Zeolite	CP	Zeo x CP	Regression
BWG	2589 ± 30	2580 ± 28*	2515 ± 23*	2487 ± 21*	2542	2580± 23	2505± 16	0.05	0.005	0.89	0.0062^A^
FI	3521 ± 35	3565 ± 39	3538 ± 53	3507 ± 41	3532	3521± 24	3544± 36	0.23	0.28	0.98	NS
FCR	1.36 ± 0.02	1.38 ± 0.02*	1.41±0.03*	1.41 ± 0.03*	1.398	1.37±0.01	1.41±0.02	0.05	0.002	0.99	0.0007^A^
**Phase 1–42 days**											
Parameter	Zeolite (%)							*p-Value*			
	0	1	2	3	Mean	Mean CP REC	Mean CP RED 3%				
								Zeolite	CP	Zeo x CP	Regression
BWG	3988 ± 65	3915 ± 45	3863 ± 45*	3836 ± 28*	3901	3921 ± 44	3881 ± 25	0.04	0.04	0.68	0.0070^A^
FI	5427 ± 50	5481 ± 61	5439 ± 77	5442 ± 46	5447	5405 ± 40	5489 ± 44	0.17	0.06	0.43	NS
FCR	1.36 ± 0.03	1.40±0.02	1.41±0.03*	1.42 ± 0.02*	1.398	1.38 ± 0.02	1.41 ± 0.02	0.05	0.04	0.94	0.0228^A^

BWG = body weight gain; FI = feed intake; FCR = feed conversion ratio; CP = crude protein; CP REC = recommended crude protein level; CP RED 3% = crude protein reduced by 3%; NS = not significant. Means marked with an asterisk (*) differ from the control treatment (Z0) according to Dunnett’s test (*P* < 0.05). A = linear effect.

During the periods from 1 to 35 days and 1 to 42 days, a decline in performance was observed when this additive was added at the last levels (2 and 3%), except in the FC of the phase from 1 to 42 days, which only differed in the conversion of the birds that ingested the diets with the maximum level of zeolite (3%).

Regarding the crude protein (CP) levels of the diets, it was found that the 3% reduction in relation to meeting the requirement harmed the GP and FC in the cumulative phases of 1 to 35 days (*P* < 0.01) and in the phase of 1 - 42 days, reducing (*P* < 0.05) the GP and FC.

### Carcass and organ yield

Carcass data is found on Table 4. The use of different concentrations of zeolite did not influence (P>0.05) the carcass and cut yield at 42 days of age, except for breast yield (*P* = 0.007), generating a quadratic effect (*P* = 0.0022). Analyzing the results through Dunnett's test, it was observed that only the diet containing 1% zeolite differed in relation to the control diet (*P* = 0.0428).

**Table 4:** Fasted live body weight, carcass weights, and cut yields (% of fasted live body weight) of broilers fed diets containing natural zeolite with or without protein reduction.

	Zeolite (%)							Mean		
Items	0	1	2	3	Mean	p-value	Regression	CP REC	CP RED 3%	p-value
FLBW(g)	3381 ± 63	3411 ± 52	3355 ± 49	3370 ± 49	3379	0.925	NS	3387 ± 40	3370 ± 32	0.76
HCW (g)	2644 ± 52	2646 ± 40	2583 ± 36	2620 ± 41	2627	0.782	NS	2639 ± 31	2604 ± 27	0.42
CCW (g)	2576 ± 51	2577 ± 39	2513 ± 35	2549 ± 46	2558	0.770	NS	2570 ± 32	2535 ± 27	0.44
Wing (%)	3.31 ± 0.03	3.26 ± 0.045	3.27 ± 0.05	3.24 ± 0.04	3.276	0.677	NS	3.29 ± 0.03	3.24 ± 0.03	0.21
Drumstick (%)	9.48 ± 0.15	9.83 ± 0.20	9.72 ± 0.16	9.53 ± 0.12	9.610	0.433	NS	9.65 ± 0.09	9.64 ± 0.14	0.93
Back (%)	17.93 ± 0.30	18.73 ± 0.30	18.33 ± 0.42	18.40 ± 0.32	18.35	0.444	NS	18.56 ± 0.20	18.08 ± 0.27	0.17
Breast (%)	30.39 ± 0.31	28.24 ± 0.50*	28.51 ± 0.47	29.32 ± 0.42	29.09	0.007^B^	Q	29.11 ± 0.34	29.12 ± 0.36	0.98
Thigh (%)	11.33 ± 0.42	11.95 ± 0.18	11.68 ± 0.20	11.58 ± 0.32	11.64	0.496	NS	11.69 ± 0.21	11.57 ± 0.21	0.69

FLBW= Fasted live body weight; HCW = Hot carcass weight; CCW = Cold carcass weight; CP REC = recommended crude protein level; CP RED 3% = 3% crude protein reduction; NS = Not significant. Q= Quadratic effect; All yields (%) are expressed relative to fasted live body weight (FLBW). Means marked with an asterisk (*) differ from the control treatment (Z0) according to Dunnett’s test (*P* < 0.05). B=Quadratic effect (Breast yield).

The use of different concentrations of zeolite did not statistically influence (P>0.05) organ yield and length of the intestine (Table 5).

**Table 5:** Average values of relative organ weights and intestinal length of broilers fed diets containing natural zeolite with or without protein reduction.

	Zeolite (%)							Mean		
Órgan (%)	0	1	2	3	Mean	p-value	Regression	CP REC	CP RED 3%	p-value
Intestinal length (cm)	201 ± 6.2	205 ± 5.2	206 ± 6.0	205 ± 3.1	205	0.93	NS	204 ± 3.6	205 ± 3.7	0.86
Intestine	3.02 ± 0.14	2.75 ± 0.09	2.95 ± 0.11	3.02 ± 0.10	2.87	0.28	NS	2.91 ± 0.06	2.97 ± 0.09	0.63
Heart	0.45 ± 0.02	0.42 ± 0.02	0.42 ± 0.02	0.42 ± 0.02	0.43	0.36	NS	0.44 ± 0.01	0.42 ± 0.01	0.17
Liver	1.78 ± 0.10	1.66 ± 0.06	1.85 ± 0.08	1.81 ± 0.07	1.78	0.44	NS	1.82 ± 0.07	1.72 ± 0.04	0.20
Abdominal fat	1.21 ± 0.13	1.37 ± 0.14	1.62 ± 0.21	1.18 ± 0.14	1.34	0.21	NS	1.30 ± 0.11	1.40 ± 0.12	0.52
Gizzard	1.68 ± 0.08	1.73 ± 0.05	1.72 ± 0.06	1.65 ± 0.06	1.69	0.79	NS	1.68 ± 0.04	1.72 ± 0.06	0.56
Pancreas	0.17 ± 0.01	0.17 ± 0.01	0.17 ± 0.01	0.18 ± 0.01	0.17	0.79	NS	0.17 ± 0.01	0.16 ± 0.01	0.35
Proventriculus	0.31 ± 0.02	0.29 ± 0.01	0.32 ± 0.02	0.32 ± 0.02	0.31	0.85	NS	0.32 ± 0.01	0.30 ± 0.01	0.58

CP REC = recommended crude protein level; CP RED 3% = 3% crude protein reduction; NS = Not significant.

### Litter quality

According to Table 6, Table 7, there was no interaction between the factors studied on the quantification parameters of ammonia and *Escherichia coli* in the poultry litter. When evaluating the zeolite levels on the quantification of volatilized ammonia in the litter, a significant effect (*P* ≤ 0.05) was observed in the periods from 1 to 35 days (*P* = 0.0170) and from 1 to 42 days (*P* = 0.0124). Through Dunnett's test (*P* < 0.05), it was verified that the diets containing levels of 2 and 3% zeolite were the ones that differed from the control diet in the phases described (Table 6). The regression analysis performed on the aforementioned phases indicated a decreasing linear effect (*P* ≤ 0.05) with the addition of zeolite starting at the 1% level (Table 8). Regarding the effect of PB levels, we found that by reducing the PB level together with amino acids, there was an increase in the amount of ammonia excreted, no matter the zeolite level.

**Table 6:** Mean values and standard deviation of ammonia quantification analyses in the growth and finishing phases of broiler chickens.

**Parameter NH_3_/mg**											
**Phase**	Zeolite (%)				Mean	Mean CP REC	Mean CP RED 3%	*p-value*			
	0	1	2	3							
								Zeolite	CP	Zeo x CP	Regression
22-35 d	0.43 ± 0.1	0.28 ± 0.05	0.24 ± 0.04*	0.22 ± 0.04*	0.29	0.22 ± 0.04	0.36 ± 0.04	0.017	0.008	0.861	0.004^A^
36-42 d	0.46 ± 0.08	0.31 ± 0.07	0.22 ± 0.04	0.21 ± 0.04*	0.30	0.26 ± 0.04	0.33 ± 0.05	0.012	0.234	0.891	0.002^A^

CP= crude protein; CP REC = recommended crude protein level; CP RED 3% = 3% crude protein reduction; NS = Not significant. Means marked with an asterisk (*) differ from the control treatment (Z0) according to Dunnett’s test (*P* < 0.05). A = linear effect.

**Table 7:** Mean *Escherichia coli* counts in litter from broilers fed diets containing natural zeolite with and without protein reduction.

**E. Coli (CFUs/g)**	Zeolite (%)				Mean		Mean CP REC	Mean CP RED 3%	*p-value*			
	0	1	2	3					Zeolite	CP	Zeo x CP	Regression
	6.11 ± 0.20	6.45 ± 0.21	6.17 ± 0.16	6.30 ± 0.30*		6.28	5.92 ± 0.10	6.60 ± 0.14	0.525	0.001	0.282	NS

CFUs: Colony forming unit; E, Coli: *Escherichia coli*; CP= crude protein; CP REC = recommended crude protein level; CP RED 3% = 3% crude protein reduction; NS = Not significant.

Means marked with an asterisk (*) differ from the control treatment (Z0) according to Dunnett’s test (*P* < 0.05).

**Table 8:** Regression equations according to variables that presented linear (L), quadratic (Q), and cubic (C) effects.

**Variable**	**Equation**	**Effect**
Body weight gain (1d – 35d)	y= -37.1x + 2635.5 R² = 0.9294	L
Feed conversion ratio (1d – 35d)	y = 0.0178x + 1.346 R² = 0.9447	L
Body weight gain (36d – 42d)	y = 50.67x + 4027.9 R² = 0.9608	L
Feed conversion ratio (36d – 42d)	y = 0.0178x + 1.3535 R² = 0.8714	L
Breast yield	y = -0.294x + 29.556. R² = 0.1544	Q
NH_3_/mg (22d – 35d)	y = -0.0673x + 0.3927 R² = 0.8132	L
NH_3_/mg (36d – 42d)	y= 0.0843x + 0.4252 R² = 0.8648	L

In microbiological analyses of the litter (Table 7), there was an isolated effect between the levels of protein in the diet (*P* ≤ 0.05), and there was no difference between the levels of Zeolite (*P* = 0.525). Diets that had reduced protein showed an increase in the count of *Escherichia coli*.

## Discussion

The use of natural zeolites in broiler diets has been the subject of debate within the scientific community, particularly due to inconsistent results regarding their effects on growth performance. While some studies report benefits related to improved feed conversion, ammonia retention, and litter quality (Karamanlis et al., 2008; Ezenwosu et al., 2022), others do not observe significant effects or report negative responses at certain inclusion levels (Eleroglu et al., 2011; Schneider et al., 2017). The response to supplementation appears to vary widely depending on factors such as zeolite purity, processing method, inclusion level, rearing conditions, and the nutritional profile of the diets. Beneficial effects have been reported with clinoptilolite inclusions below 1% in broiler diets (Tatar et al., 2012), as well as with levels above 2% and 3% (Nikolakakis et al., 2013; Pavlak et al., 2022), highlighting the lack of consensus regarding a standardized dose–response relationship for natural zeolite supplementation. Consequently, the current literature remains heterogeneous, emphasizing the need for further studies that consider the interaction between inclusion levels, zeolite chemical composition, and different nutritional contexts. This lack of standardization makes it difficult to define consistent responses to zeolite supplementation, particularly when associated with crude protein reduction strategies. In this context, evaluating not only inclusion level but also its interaction with dietary protein content becomes relevant to better understand potential synergistic or antagonistic effects on broiler performance and physiological traits.

Based on the results of the present study, zeolite inclusion negatively affected feed conversion, weight gain, and final body weight during the 1–35 and 1–42 d periods. These effects were primarily associated with reductions in body weight observed in these phases. Only diets containing higher zeolite levels (2 and 3%) differed from the control diet. These findings agree with those of Abdelrahman et al. (2023), who evaluated zeolite inclusion at 1, 2, and 3% and reported that broilers fed 3% zeolite exhibited significantly lower weight gain and final body weight (*P* < 0.05) compared with other treatments. In contrast, Qu et al. (2019) reported that broiler diets containing up to 2% zeolite promoted increased body weight and weight gain. However, Pavlak et al. (2022) suggested that zeolite inclusion above 1% may interfere with the protein–energy balance and cation–anion equilibrium of the diet, thereby negatively affecting growth performance. This trend was confirmed in the present study, particularly in diets formulated with marginal protein levels, in which 2 and 3% zeolite resulted in a marked reduction in growth performance. These findings reinforce the need for caution when using zeolite at high inclusion levels, especially in association with protein-restricted diets.

It is possible that the zeolite levels evaluated in this study contributed to the observed performance impairments. Similar results were reported by Abdelrahman et al. (2023), who concluded that zeolite inclusions above 10 g/kg of feed (1%) negatively affected broiler performance. Likewise, Šuchý et al. (2006) evaluated 1 and 2% zeolite supplementation and concluded that high doses of clinoptilolite (2%) in young broilers (up to 30 d of age) may exert a suppressive effect on growth performance, suggesting that inclusion levels should be adjusted according to the birds’ developmental stage.

Conversely, Abdelrahman et al. (2023) observed a significant improvement (*P* < 0.05) in feed conversion rate in broilers fed diets containing 1 and 2% zeolite at 5 and 6 wk of age compared with the control diet. These authors attributed the improvement to mechanisms previously described in the literature, such as reduced digesta passage rate, enzyme immobilization, and modulation of intestinal microbiota (Hcini et al., 2018), which may enhance digestion and nutrient absorption.

Several studies have evaluated zeolite inclusion in broiler diets, reporting variable responses depending on dosage. At inclusion levels up to 1%, improvements in growth performance have been observed by some authors. Mallek et al. (2012) reported that broilers fed 1% zeolite achieved higher body weight at 45 d of age (2.44 vs. 2.24 kg; *P* < 0.001) compared with non-supplemented birds. Wawrzyniak et al. (2017) also observed increased weight gain in broilers supplemented with 2 and 3% zeolite, suggesting enhanced digestive enzyme secretion and improved nutrient digestibility and intestinal health. In contrast, other studies reported no significant effects of 1% zeolite inclusion on production parameters (Waldroup et al., 1984; Elliot et al., 1991; Banaszak et al., 2020).

At inclusion levels above 1%, results become even more controversial. Khademi (2003) reported improved performance with zeolite inclusions up to 7%, whereas higher levels resulted in adverse effects. Schneider et al. (2017) suggested an optimal inclusion of 0.5%, while Vieira et al. (2023) recommended 0.93% clinoptilolite in commercial laying hen diets without compromising productive performance or egg quality. Positive results with higher zeolite levels (2 to 6%) were reported by Karamanlis et al. (2008) and Basha et al. (2016). Conversely, Khademi (2003) and others highlighted performance impairments associated with excessive zeolite inclusion.

Collectively, these findings indicate that broiler responses to zeolite supplementation are dose-dependent, with lower inclusion levels (up to 1%) generally being more favorable, whereas higher levels (>2%) may induce adverse effects, possibly due to alterations in gastrointestinal tract characteristics, such as pH and ionic composition of digestive fluids (Martin-Kleiner et al., 2001).

Regarding dietary crude protein (CP) content, a 3% reduction relative to the recommended level impaired weight gain and feed conversion in the later production phases (1–35 and 1–42 d). These results demonstrate that reducing dietary CP by 3% is not feasible when essential amino acids are simultaneously reduced below requirement levels. Adequate amino acid supplementation is essential when formulating low-protein diets to maintain growth performance. Diets with reduced CP accompanied by proportional reductions in amino acids can negatively affect broiler performance (Belloir et al., 2017; Cho et al., 2023; Aderibigbe et al., 2024).

Notably, no interaction between zeolite inclusion and dietary protein level was observed in this study, indicating that protein reduction did not modify the response to zeolite supplementation. Olver (1989) evaluated the inclusion of 5% clinoptilolite in laying hen diets with and without protein reduction and reported improved productivity with zeolite supplementation, although negative effects were observed in low-protein diets (13% CP) compared with diets containing 16% CP.

Regarding carcass and organ yield, a negative effect on breast yield was observed at the 1% zeolite inclusion level, whereas other variables were not significantly affected. This finding contrasts with results reported by Palic et al. (1993) and Safaei Katouli et al. (2012), who observed increased meat yield, particularly breast and thigh percentages, with zeolite supplementation. These authors attributed such improvements to enhanced gastrointestinal health and feed efficiency. However, in the present study, the absence of performance benefits and the reduction in breast yield at 1% zeolite suggest that broiler responses may depend on inclusion level, treatment duration, or specific characteristics of the zeolite used.

Other studies have also reported no significant effects of zeolite on carcass yield. Safameher (2008) observed no changes with 2% zeolite inclusion, while Ozturk et al. (1998), Moghaddam et al. (2005), Khajali et al. (2006), and Tatar et al. (2012) reported no effects even with higher inclusion levels.

Conversely, Ortatatli and Oguz (2001) reported reduced liver yield at 21 d of age in broilers fed 2.5% clinoptilolite compared with 1.5%. Fisinin et al. (1985) observed increased carcass yield with 5% zeolite inclusion, whereas Le Gall-David et al. 2016 reported increased intestinal length and yield in broilers supplemented with Cu-bound zeolite.

Litter quality represents a major limitation in poultry production. Among the factors affecting litter quality, moisture, microbial population, and ammonia (NH₃) emissions are particularly critical, as they directly influence broiler performance, carcass yield, and respiratory and intestinal health. Elevated atmospheric ammonia concentrations impair performance and increase disease susceptibility (Beker et al., 2004), in addition to causing environmental and economic losses. Zeolites have been proposed as an alternative strategy to control ammonia and moisture due to their adsorption capacity and water retention properties (Schneider et al., 2016).

In the present study, ammonia emission was progressively reduced with zeolite inclusion from 1% onward, regardless of dietary protein level. Reductions of 36, 44, and 49% were observed during the 1–35 d period, and 34, 53, and 54% during the 1–42 d period, for 1, 2, and 3% zeolite inclusion, respectively. Similar findings were reported by Elsherbeni et al. (2024), who observed gradual reductions in litter ammonia when zeolite was added either to the diet or directly to the litter.

When evaluating the nitrogen content in the litter, Tatar et al. (2012) observed a significant decrease, due to dietary supplementation of 4% zeolite at 42 days of age (*P* < 0.05). Pavlak et al. (2023) describe that chickens fed diets containing zeolite at levels of 0.5 and 1.0% presented lower concentration in ammonia levels compared to the control group only at 24 days of age. This attenuating effect on ammonia emissions can be justified by the fact that zeolite enhances the use of nutrients more efficiently, in terms of the adsorption of larger quantities of protein nitrogen. Although reduced crude protein diets are often associated with lower ammonia emission, in the present study the reduction in dietary protein was not accompanied by additional amino acid supplementation, which may have compromised nitrogen utilization efficiency in broilers. As a consequence, greater nitrogen excretion may have occurred, contributing to the increased ammonia emission observed.

In addition, litter characteristics, such as higher moisture retention and increased microbial activity, may have influenced the ammonia volatilization process, thereby intensifying this effect. In this context, reduced-protein diets in which digestible amino acids follow the same downward trend may result in undesirable outcomes, as aminos acid imbalance can stimulate muscle protein degradation as a compensatory mechanism. This process may, at least in part, explain the results observed in the present study.

Diets containing 3% reduced protein resulted in an increase in the ammonia concentration in the litter when compared to the control diet. It is known that excess protein or an imbalance between amino acids can compromise the productivity of broilers, due to the generation of an excessive load of amino acids in the bloodstream which, in order to be metabolized, require an extra expenditure of energy that is diverted from production to the processes of nitrogen excretion in the form of uric acid (Aletor et al., 2000). However, low-protein diets are effective in reducing nitrogen excretion into the environment (Bregendahl et al., 2002), but they must contain digestible amino acids in adequate amounts.

In disagreement, when collecting the excreta of broilers and quantifying the total nitrogen content, Schneider et al. (2017) did not find a significant effect in diets with 0.5% zeolite, however, they found a reduction in the humidity of the excreta, not a reduction in the nitrogen content in the excreta. Safaei et al. (2012) observed only a reduction in the humidity of the excreta when testing 3% zeolite in chicken feed. Nikolakakis et al*.* (2013) reiterate that levels of 2 and 3% zeolite altered the moisture levels of the excreta, but did not influence the amount of ammonia volatilized in the excreta. Like the previous authors, Pavlak et al. (2023) found no differences when testing levels of 0.5% and 1% of zeolites in the diets.

When evaluating higher zeolite levels (3.1, 6.3 and 12.5 kg/m2), Li et al. (2008) obtained significant results regarding the reduction of ammonia in the excreta of 36, 62 and 92% for each inclusion level, respectively. However, this study included zeolite in two forms, in the diet and directly incorporated into the litter, which may justify the significant result on the evaluated parameter, unlike the experiments with dietary inclusions of zeolite.

Some studies suggest that including clinoptilolite in the diet is not the best way to reduce ammonia levels in litter because the reduction of the ion (NH_3_) in litter is more visible with the direct addition of the mineral to the litter. Reductions in moisture and pH are more frequently obtained when zeolites are included in broiler diets (Safaei et al., 2012; NikolakakiS et al., 2013). Still Elsherbeni et al*.* (2024), assert that the improvement in terms of litter quality is due to the different zeolite treatments, suggesting the addition of these to the diet and directly to the litter. However, the results of the present study indicate that the inclusion of zeolite in the diets has an efficient action in broiler chickens, with regard to the reduction of ammonia.

In the evaluation of the microbiological count of *Escherichia coli* in the litter, the levels of natural zeolite in the diet did not show statistical differences in this research. These results may be related to the origin of the zeolites, chemical composition, presence of impurities or manner of use. However, an increase in the quantity of *E. coli* was observed in the litter of the birds that consumed diets containing 3% protein reduction. This situation may be justified by the influence of this diet on the harmfully increased levels of ammonia because the excreted nitrogen compounds present in poultry litter are rapidly decomposed by bacteria and fungi (Hernandes et al. 2001) that use uric acid to synthesize their own proteins and the excess nitrogen is released in the form of ammonium (NH^4+^) or ammonia (NH_3_) (Gonzáles & Saldanha, 2001).

In an in vitro study with *Escherichia coli* and *Salmonella Typhimuriumhe*, Wu et al. (2013) observed that the microbiological effect of litter was adsorbed by clinoptilolite, but there was no general statistical bactericidal effect. A study has also been published in which chemical modification of clinoptilolite with organic acids (e.g. formic acid) resulted in increased hydrophobicity of the mineral surface, increased bactericidal effect against *Escherichia coli* and its toxins, and increased cation exchange capacity (Uchida et al., 1992; Daković et al*.*, 2005).

Microbiological suppression at the intestinal level is reflected in this count, considering the level of excretion of this pathogenic microbiota in the litter. Therefore, zeolites have a strong adsorption capacity and can adsorb toxic substances and bacteria in the intestine that are harmful to the host, eventually excreting them from the animal's body (Slamova et al., 2011; Wu et al., 2013; Zhou et al., 2014).

The most significant effects of natural zeolites may be due to chelating properties or an intrinsic mechanism in the body that could lead to a reduction in litter moisture and the microbiological flora of the bird. The addition of 2% zeolite to broiler feed reduced litter moisture (Gezen et al., 2004) decreased the organic content of the litter, and improved its quality (Karamanlis et al., 2008). Zeolite can adsorb intestinal pathogenic bacteria, prevent damage, reinforce the intestinal mucosal barrier, and help in the regeneration of the epithelium (Tatar et al., 2008; Caflisch et al., 2018).

The results analyzed in this research diverge from those observed by Le gall-david et al*.* (2016), in which diets containing zeolite significantly reduced the diversity of bacterial phyla present in the small intestine of broilers, and this depletion led to a predominance of *Firmicutes* (ca. 99%) and an elimination of pathogenic genera such as *Enterococcus, Shigella*, or *Escherichia*. The same authors conclude that zeolite acts by selecting and occasionally suppressing the growth of pathogens or promoting the growth of *Lactobacillus* species, supporting a healthy digestive system through the "competitive exclusion" of pathogenic bacteria.

The mechanisms behind the observed effects may be related to the adsorption and ion exchange capacity of clinoptilolite, which influence nutrient digestibility and absorption (Reis et al., 2020). Although the present study did not find significant suppression of *E. coli* populations in the litter, it is important to note that studies such as those by Daković et al. (2005) and Hrenovic et al. (2012) show that chemical modification of zeolite can enhance its antimicrobial properties. This variation in properties and results suggests that zeolite processing and purity are critical factors for its zootechnical effect.

As previously reported, there is a wide variety of zeolites, their composition, chemical proportion, and the ions and cations they carry, which can directly influence the type of adsorption to which it is subjected. *In vitro* studies have shown that clinoptilolite can adsorb *Escherichia coli*, but without bacteriostatic or bactericidal effects (Ramu et al., 1997). For example, zinc-loaded zeolite showed better antibacterial activity for *E. coli* than copper-loaded zeolite (Hrenovic et al., 2012). This information may justify the results, given that the zeolite used in this study had equivalent proportions of copper (Cu) and zinc (Zn).

The result of this diversity of zeolites that have different chemical compositions, and which reflect their biological behavior in the animal organism, will consequently generate scientific publications with divergent results. There is continuous progress in research, in order to attest the adequate dose of these minerals and which parameters in poultry production are effectively viable in their use. Katouli et al. (2010) suggest that the differences found in the results reported in literature can be explained due to the structure of the mineral and the content of metal oxides that can differ even among natural zeolites depending on extrinsic factors such as geographic-environmental factors. Eleroglu et al. (2011) conclude that experiments with different types of natural zeolites in different elemental proportions of Si/Al+Fe, alkali/alkaline earth, Na/K are necessary to better understand their influence on the zootechnical parameters of broiler chickens.

In this context, further research will be necessary to improve the understanding of the mechanisms by which natural zeolites act on animal metabolism, with the purpose of efficiently delimiting the significant levels of inclusion of these minerals in poultry diets. In this logic, scientific production must follow a homogeneous description of the characteristics of these minerals in the respective tests. This aspect is particularly relevant, since the lack of detailed information on the origin, type of processing, purity content, impurities and chemical composition of zeolites in the reviewed studies can directly impact the results obtained, contributing to the discrepancy of data found in literature.

## Conclusion

Under the conditions of this study, the dietary inclusion of up to 1% natural clinoptilolite zeolite effectively reduced ammonia emissions without compromising growth performance, carcass yield, or relative organ weight, regardless of dietary crude protein level. In contrast, a 3% reduction in crude protein without adequate amino acid adjustment impaired broiler performance, particularly when associated with higher zeolite inclusion levels (2 and 3%). These results indicate that moderate zeolite inclusion combined with nutritionally adequate protein levels presents greater nutritional and environmental viability. Further studies should investigate the interaction between zeolite composition and industrial amino acid supplementation strategies to optimize the use of this additive in poultry production.

## CRediT authorship contribution statement

**Camila Guedes Valadares:** Writing – review & editing, Writing – original draft, Supervision, Project administration, Methodology, Investigation, Formal analysis, Data curation, Conceptualization. **Maria do Carmo Mohaupt Marques Ludke:** Writing – review & editing, Writing – original draft, Validation, Resources, Project administration, Methodology, Investigation, Funding acquisition, Formal analysis, Data curation, Conceptualization. **Jorge Vitor Ludke:** Writing – original draft, Methodology, Funding acquisition, Formal analysis, Data curation, Conceptualization. **Carlos Bôa-Viagem Rabello:** Methodology, Formal analysis, Data curation, Conceptualization. **Gláucia Manoella de Souza Lima Gomes:** Methodology, Data curation, Conceptualization. **Dayane Albuquerque da Silva:** Writing – review & editing, Investigation, Formal analysis, Data curation, Conceptualization. **Apolônio Gomes Ribeiro:** Writing – review & editing, Formal analysis, Data curation, Conceptualization. **Arlei Coldebella:** Software, Data curation, Conceptualization. **Esterfani Pereira da Silva:** Formal analysis, Data curation, Conceptualization. **Marcela de Araújo Sobral:** Methodology, Data curation, Conceptualization. **Kananda Rohhden dos Santos:** Data curation, Conceptualization. **Clener Manoel Albino Fausto:** Data curation, Conceptualization. **Lucas Rannier Ribeiro Antonino Carvalho:** Writing – review & editing, Data curation, Conceptualization.

## Disclosures

The authors declare no conflicts of interest.
